# Transcriptional foliar profile of the C_3_-CAM bromeliad *Guzmania monostachia*

**DOI:** 10.1371/journal.pone.0224429

**Published:** 2019-10-29

**Authors:** Helenice Mercier, Maria Aurineide Rodrigues, Sónia Cristina da Silva Andrade, Luiz Lehmann Coutinho, Bruno Nobuya Katayama Gobara, Alejandra Matiz, Paulo Tamaso Mioto, Ana Zangirolame Gonçalves

**Affiliations:** 1 Departamento de Botânica, Instituto de Biociências, Universidade de São Paulo, São Paulo, Brazil; 2 Departamento de Genética e Biologia Evolutiva, Instituto de Biociências, Universidade de São Paulo, São Paulo, Brazil; 3 Departamento de Zootecnia, Escola Superior de Agricultura “Luiz de Queiroz”, Universidade de São Paulo, São Paulo, Brazil; 4 Departamento de Botânica, Centro de Ciências Biológicas, Universidade Federal de Santa Catarina, Santa Catarina, Brazil; Consiglio Nazionale delle Ricerche, ITALY

## Abstract

*Guzmania monostachia* is an epiphytic tank bromeliad that displays the inducible CAM photosynthesis under stressful conditions and had the highest stomata density in the leaf apex, while the base portion has the highest density of trichomes, which are specialized structures used to acquire water and nutrients from the tank solution. In order to correlate the genetic factors behind these morpho-physiological characteristics along the leaf blade of *G*. *monostachia*, a comparative transcriptome analysis was performed to identify the functional enriched pathways and unigenes that could play a role in the apical, middle and basal leaf portions. A total of 653 million reads were used for *de novo* transcriptome assembly, resulting in 48,051 annotated unigenes. Analysis of differentially expressed genes (DEGs) among distinct leaf regions revealed that 806 DEGs were upregulated in the apex compared to the middle portion, while 9685 DEGs were upregulated in the apex and 9784 DEGs were upregulated in the middle portions compared to the base. Our outcomes correlated some DEGs and identified unigenes with their physiological functions, mainly suggesting that the leaf apex was related to the regulation of stomatal movement, production of chlorophyll, cellular response to stress, and H_2_O_2_ catabolic process. In contrast, the middle portion showed DEGs associated with the transport of amino acids. Furthermore, DEGs from the leaf base were mainly correlated with responses to nutrients and nitrogen compounds, regulation of potassium ion import, response to water deprivation, and trichome branching, indicating that, at least in part, this leaf portion can replace some of the root functions of terrestrial plants. Therefore, possibly candidate unigenes and enriched pathways presented here could be prospected in future experimental work, opening new possibilities to bioengineer non-inducible CAM plants and/or improve the fertilization use efficiency by increasing leaf nutrient acquisition of crop plants.

## Introduction

*Guzmania monostachia* is an epiphytic tank-forming bromeliad that has been used as a model-plant for photosynthetic studies as it up-regulates the Crassulacean acid metabolism (CAM) depending on the environmental conditions [1–3]. For instance, under water shortage or high light intensity, the CAM syndrome can be induced in the leaves. However, after the suspension of the stressful condition, the C_3_ mode can operate again [1]. As other epiphytic bromeliads, *G*. *monostachia* has a reduced root system while the leaves have great importance for plant metabolism. Besides the photosynthesis *per se*, the leaves replace at least in part the root function related to water and nutrient uptake, since the leaf base portion has the highest density of trichomes, which are specialized structures used for tank solution absorption, while the apical portion has the largest number of stomata [4,5]. Therefore, there are clear morphological differences along the leaf blade.

Besides the morphological differences, the distinct foliar portions also present physiological differences. Considering that bromeliads have a rosette shape, the apical portion of the leaves receives the lightest incidence compared to the middle and basal portions. Thus, at the same time the leaf apex of *G*. *monostachia* presents the highest up-regulation of CAM, it is also able to increase the reactive oxygen species (ROS) scavenging system that includes antioxidant enzymes and carotenoids in response to water shortage [6]. Recently, other research performed with *G*. *monostachia* also showed that the ammonium nutrition had a positive impact on CAM expression in the apex portion as a more efficient osmotic adjustment, and malate transport to the vacuole was stimulated under this nitrogen source associated with water deficit [7].

Since morphological and physiological differences were found in the leaf blade of *G*. *monostachia*, the present study sought to investigate the transcriptional profile of the leaf portions of this epiphytic bromeliad. To our knowledge, this is the first transcriptome sequencing of a Tillandsioideae species whose traits are considered the most derived within the Bromeliaceae family. The first transcriptional profile data of a bromeliad refers to the pineapple (*Ananas comosus* var. comosus) that was performed from a ripe yellow fruit [8]. After that, other RNA sequencing (RNA-seq) was obtained from *Ananas comosus* using leaves, roots, and stems from the ornamental variety bracteatus [9], and from the flowering-induced buds of the var. smooth cayenne [10]. The Bromelioideae species *Aechmea fasciata* [11] and two Pitcairnioideae species, *Pitcairnia albiflos* and *P*. *staminea* [12], also had their transcriptome analyzed. For the ornamental bromeliad *A*. *fasciata*, shoot tip tissues of juvenile and adult plants were harvested to compare flower development induced by ethylene treatment, while mature flowers and leaves of *Pitcairnia* were compared to comprehend the speciation process between these two species.

The leaf blade of tank bromeliads present morpho-functional heterogeneity with a gradient of specializations from the basal to the apical portions [3–7, 13–15], but in a few cases the morpho-physiological aspects are systematically investigated in relation to their functional significance [16] and, to our knowledge, nothing is known about the genes that could play a key role in each leaf portion. In order to provide an overview of the transcriptional profile of the leaf blade of *G*. *monostachia*, we collected leaf samples of bromeliads grown in controlled conditions to obtain a robust comparison on the molecular functions of each leaf portion: apex, middle, and base. Our research brings new data on the differentially expressed genes (DEGs) and enriched pathways comparing the chlorophyll leaf portion (apex and middle) and the basal one. We highlight the correlation between some DEGs and identified unigenes with their physiological functions, suggesting the base as responsible for nutrient uptake and response to nitrogen compounds as well as the apex and middle portions can perform photosynthesis and oxidoreduction reactions. Our results could be a useful resource for bioengineering economically essential plants since we have shown unigenes and enriched pathways that can be explored in the future to increase, for example, their leaf absorbing capacity.

## Materials and methods

### Ethics statement

This study was conducted according to relevant national and international guidelines. Record number A279903 obtained from the Brazilian Genetic Diversity bank (SisGen), *Ministério do Meio Ambiente*, Brazil, for *Guzmania monostachia* (L.) Rusby ex Mez var. *monostachia*.

### Plant material

*Guzmania monostachia* were obtained by *in vitro* propagation and were transferred to pots when atmospheric plants achieved nearly 30 mm long as described in detail in Rodrigues *et al*. [15]. After, adult plants (about 2.5 years) were maintained under controlled conditions in a growth chamber for 1 month with a 12-h photoperiod, an average photosynthetic photon flux density of 250 μmol m^-2^ s^-1^, and day/night values for air temperature and relative humidity of 27/22°C and 60/70%, respectively. During this period, plants received distilled water daily, and half of them received a complete nutrient solution every 15 days (half of the concentration of macronutrients from Knudson [17] and micronutrients from Murashige and Skoog [18]), while the other half received no nutrients. After the 30 days, the plants were submitted to three distinct treatments (n = 3 replicates per treatment): (i) bromeliads that received nutrients previously were subjected to seven days of drought (drought_nutrients), and bromeliads that did not receive nutrients were subjected to (ii) drought (drought_without nutrients), and (iii) received distilled water for seven days (water_without nutrients). Subsequently, the 8^th^ to 12^th^ leaves from the innermost nodes of the plants were harvested at 7 am and 7 pm, divided into apical, middle, and basal portions, frozen immediately with liquid nitrogen, and stored at -80°C until the RNA extraction. *Guzmania monostachia* plants were submitted to different treatments of water and nutrition and were sampled at distinct times (7 am and 7 pm) in order to provide a large pool of mRNAs transcribed along the leaf blade, evidencing remarkable differences among each leaf portion (n = 18 replicates per leaf portion).

### RNA extraction, quality control and sequencing

RNA was extracted from 100 mg of frozen leaf material powdered with liquid nitrogen using Trizol^®^ reagent (Invitrogen, USA) and the PureLink^®^ RNA Mini Kit (Ambion) according to the manufacturer’s protocol. Total RNA concentration and quality (260/280 nm = 2.0 ± 0.1) were checked with the NanoDrop^®^ 2000 Biophotometer (Thermo Fisher Scientific, USA) and considered for sufficient purity. RNA integrity was verified with 1% agarose gel by electrophoresis and using the RNA 6000 Nano LabChip Kit and a Bioanalyzer 2100 (Agilent Technologies Inc., USA). Samples of RNA integrity number ≥ 6.5 were pooled to generate libraries with the TruSeq RNA Sample Preparation Kit, Set A (Illumina Inc., USA). Paired-end sequences (2 x 125 bp) were generated via the HiSeq 2500 (Illumina HiScanSQ platform) at the Centro de Genômica Funcional Aplicada a Agropecuária e Agroenergia, ESALQ, USP, Piracicaba, São Paulo, Brazil.

### De novo assembly and functional annotation

The generated sequences were filtered to remove low-quality sequences and contaminated reads using the SeqyClean 1.9.9 (https://github.com/ibest/seqyclean), and only high-quality paired-end sequences (with average PhredScore over 24) were used for further analysis. Sequence normalization was performed with the Trinity software package using the normalize_by_kmer utility with 30 defined as the maximum coverage [19]. *De novo* transcriptome assembly was performed using Trinity (assembly by short sequences) [19, 20]. We used CD-hit [21] to filter out redundant sequences with similarity over 95%. Reads are available at the Bioproject ID PRJNA532595.

Using the tool tblastx from the BLAST suite [22], the *G*. *monostachia* transcripts were annotated using the database sequences from Viridiplantae (taxa ID 33090, VP) and Monocotyledons (taxa ID 4447, MC). The e-value threshold was set at 1e^-10^ with 125 bp as the minimum alignment. Functional annotation was performed using the Blast2GO based on the BLAST results (www.blast2go.com; [23]).

### Differential expression analysis

The 54 samples (18 replicates per leaf portion) were mapped against the 48,051 annotated contigs using Bowtie2 [24]. Leaf portions’ (apex, middle, and base) and bromeliad treatments’ (drought_nutrients, drought_without nutrients, and water_without nutrients) pairwise comparisons were performed to identify DEGs, and the significance of differential gene expression was assessed with the DESeq2 program [25]. These analyses were run in R/Bioconductor [26] and each comparison analyses were conducted separately. Count data were first normalized using logarithmic scale for the negative binomial GLM for differences in sequencing effort and proportionality across libraries, while common dispersions were calculated using the plotDispEsts() function. The multi-factor design was employed, in which the treatments were considered as the variable conditions within each leaf portion. Gene-wise exact tests for differences in the means between two groups of negative-binomially distributed counts were then computed, and the Benjamini and Hochberg false discovery rate (FDR) correction was applied adjusting to the *P* value. Gene Ontology (GO) functional enrichment analysis of DEGs was performed by Fisher’s exact test (FDR < 0.05) using the Blast2GO [23].

## Results

### Sequencing and de novo assembly

The RNA-seq of *Guzmania monostachia* was constructed based on RNA extracted from 54 samples of leaves divided into three distinct portions (apex, middle, and base). In total, about 752 million reads were produced from the mRNA libraries, and the average income per library was about 15 million reads (Table 1, S1 Table). The low-quality and contaminated reads were removed using the SeqyClean 1.9.9 (https://github.com/ibest/seqyclean), and about 653 million reads (87%) were used for the assembly (Table 1, S1 Table). The *de novo* assembler Trinity [19, 20] produced 94,081 contigs after been filtered out using the CD-hit [21].

**Table 1:** Assembly of *Guzmania monostachia*. Summary of the assembly and annotation of *Guzmania monostachia*.

	**Number of sequences (%)**
Total number of reads	752,981,826
Total number of reads used for assembly	653,859,428 (87%)
Total number of contigs	94,081
Mean length of contigs	1,016.9
Unigenes with annotation	48,051

### Functional annotation

**Fig 1:**
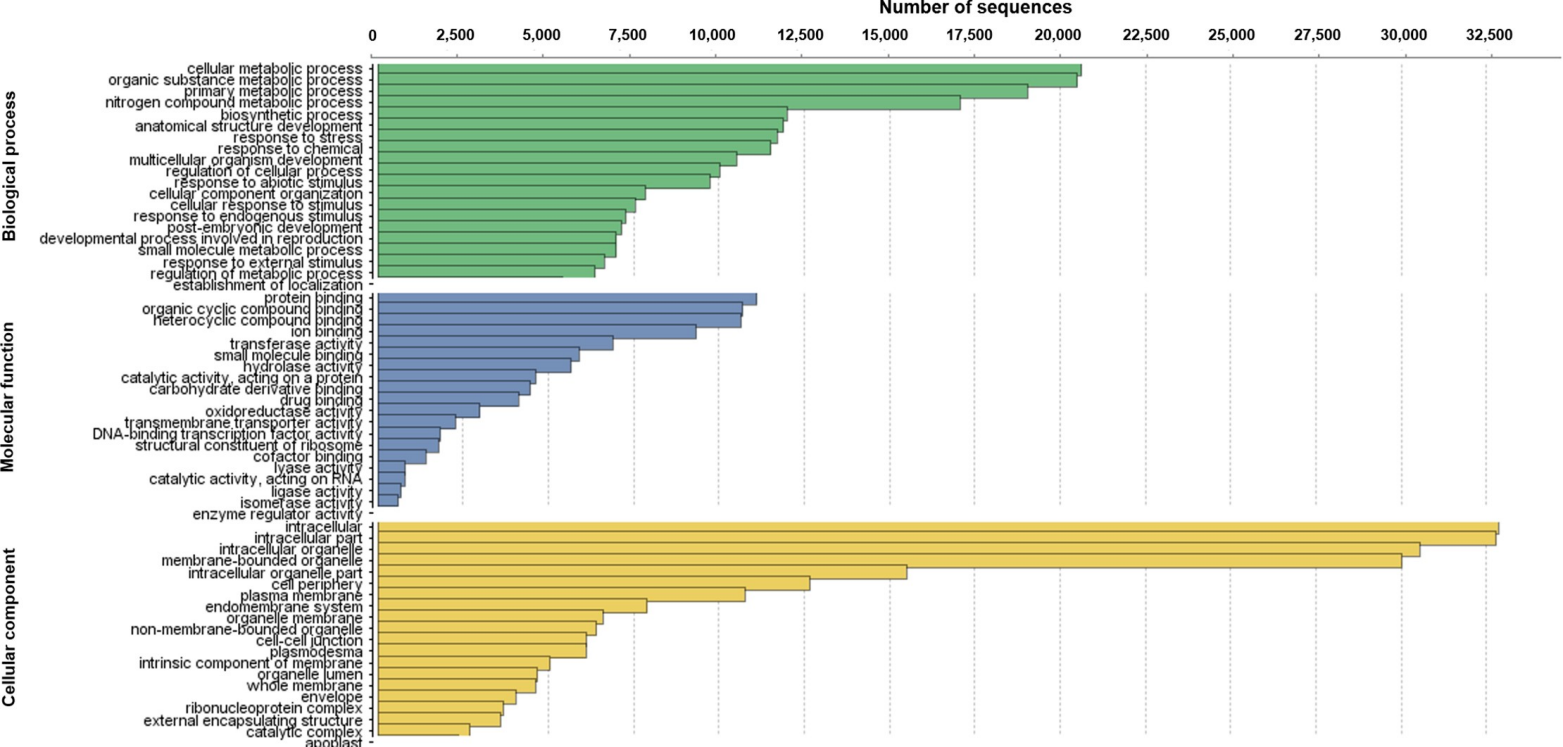
GO terms of the leaf transcriptome of *Guzmania monostachi*a. Functional annotation and comparison of the second-level Gene Ontology (GO) terms of the leaf transcriptome of *Guzmania monostachia*.

The unigenes were annotated using the BLAST suite [22], resulting in 48,051 annotated sequences (Table 1) and were classified into 53 categories in the three main gene ontology terms (GO level 2): biological process, molecular function, and cellular component (Fig 1). The highest number of sequences represented the ‘cellular metabolic process’, ‘organic substance metabolic process’, and ‘primary metabolic process’ for the biological process category; ‘protein binding’ and ‘organic cyclic compound biding’ for the molecular function category; and ‘intracellular’, ‘intracellular part’, and ‘intracellular organelle’ for the cellular component category (Fig 1).

### Differential expression analysis

**Fig 2:**
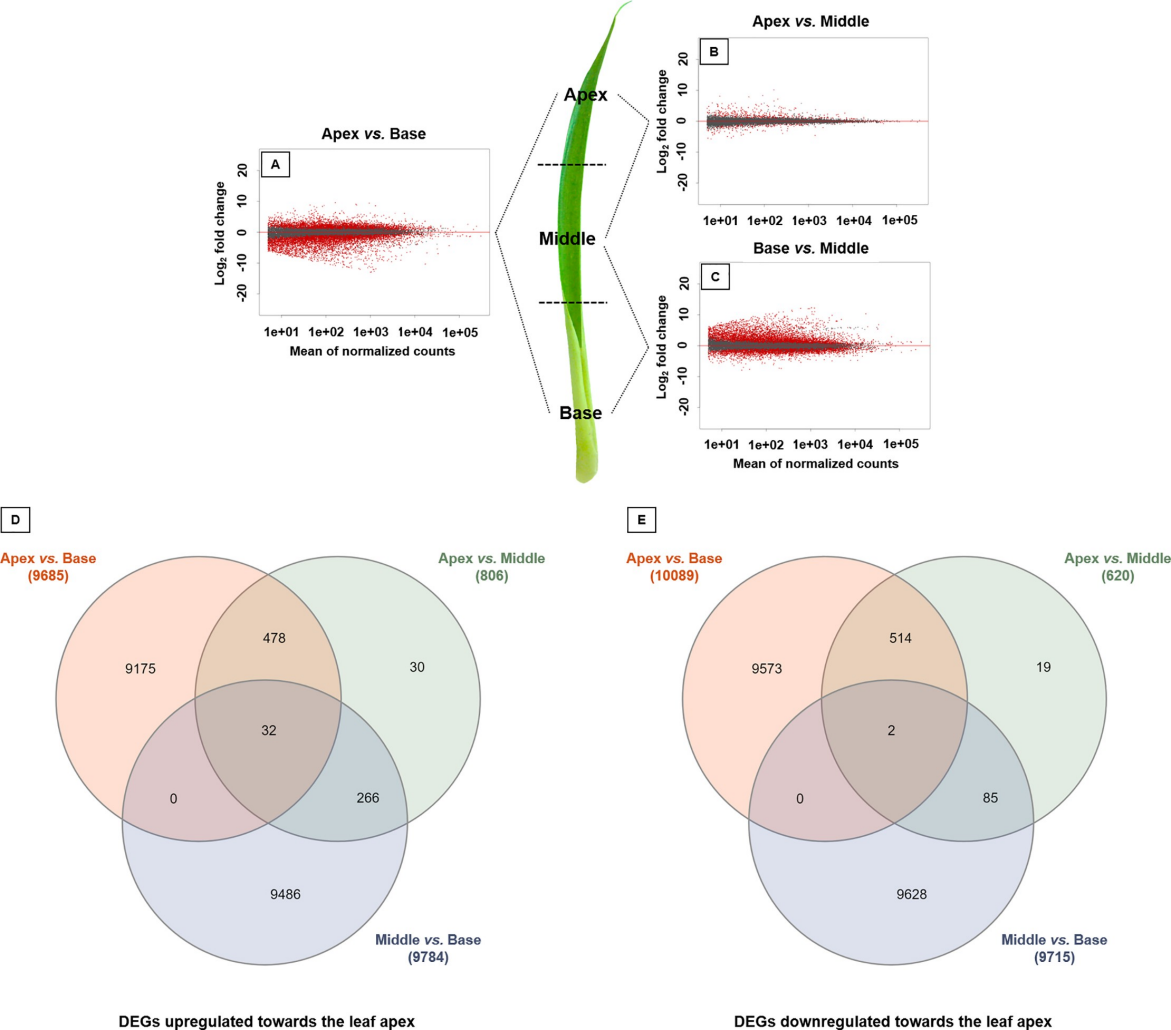
DEGs along the leaf blade of *Guzmania monostachia*. Transcriptional profiles and patterns of differentially expressed genes (DEGs) along the leaf blade of *Guzmania monostachia*. Differentially expressed genes between **(A)** apex and base, **(B)** apex and middle, and **(C)** middle and base portions are shown. Differentially expressed genes are in red, and those with no differentiation are in black. Venn diagrams representing the number and percentage of unigenes (**D**) upregulated towards the leaf apex and (**E**) downregulated towards the leaf apex.

We verified that the transcriptional profiles differed among leaf portions. Herein, the apex showed higher number of DEGs than the leaf base (Fig 2A), but was quite similar when compared to the middle leaf portion (Fig 2B), while the middle leaf portion showed higher DEGs than the leaf base (Fig 2C). The number of DEGs and unigenes upregulated and downregulated in the chlorophyll portion compared to the leaf base were presented in Fig 2D and 2E. In total, 19774 DEGs were observed comparing apex and base, in which 9685 were upregulated in the apex and 10089 were upregulated in the base. Comparing the apex and middle portions, 806 DEGs were upregulated in the apex and 620 were upregulated in the middle (total of 1426 DEGs). Finally, a total of 19499 DEGs were observed comparing middle and base, in which 9784 were upregulated in the middle portion and 9715 were upregulated in the base (Fig 2D and 2E).

**Fig 3:**
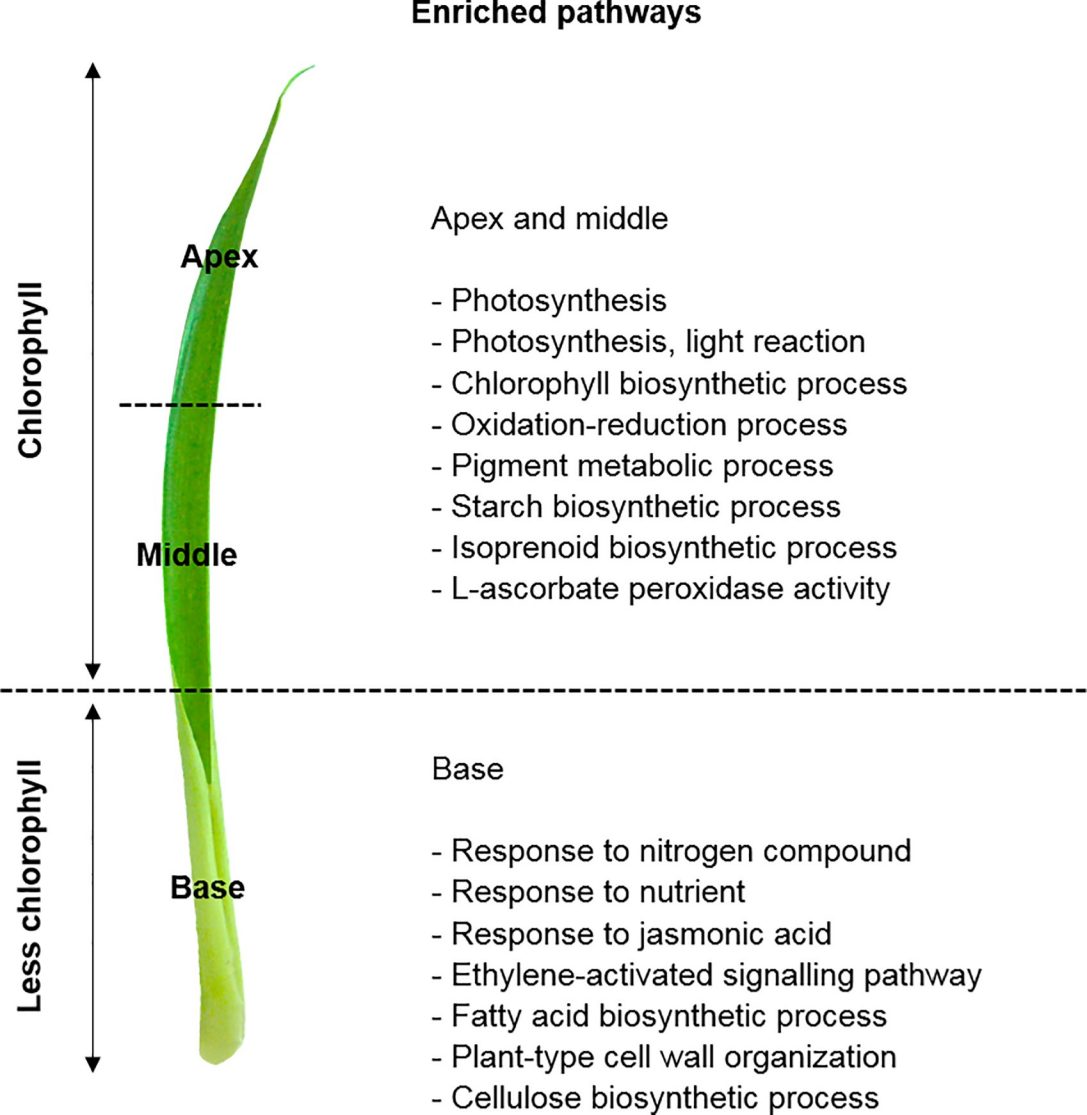
GO and DEGs of the chlorophyll *vs*. less chlorophyll leaf portions. Gene Ontology (GO) functional enriched pathways within differentially expressed genes (DEGs) between the chlorophyll leaf portion (apex and middle) compared to the less chlorophyll portion (base) of *Guzmania monostachia*. This figure was constructed based on the enriched pathways shown in S3 and S4 Tables (Supporting information), and according to the GO terms for biological process, cellular component, or molecular function categories, which showed differential abundance by Fisher’s exact test (FDR < 0.05).

In order to evaluate the biological functions performed by the DEGs identified in the comparisons between each leaf portion, we performed the functional enrichment analysis based on the GO annotation provided by the Blast2GO. The most highly represented GO categories within each leaf portion comparisons showed that the chlorophyll leaf portion (apex and middle) displayed functions associated with photosynthesis (phosphoenolpyruvate carboxylase; *PEPC1*), photosynthesis light reaction, chlorophyll (*CHLG*), starch (*SSY1*) and isoprenoid biosynthetic processes, oxidoreduction reactions, pigment metabolic process, and L-ascorbate peroxidase activity (*APX4*) (S2–S4 Tables; Fig 3). On the other hand, the less chlorophyll leaf portion (leaf base) displayed functions related to responses to nitrogen compound (*NRT1*.*1*) and nutrients, fatty acid and cellulose (*CESA9*) biosynthetic processes, plant-cell wall organization, ethylene-activated signaling pathway (*ACCO1*), and response to jasmonic acid (*LOX4*) (S2–S4 Tables; Fig 3).

**Fig 4:**
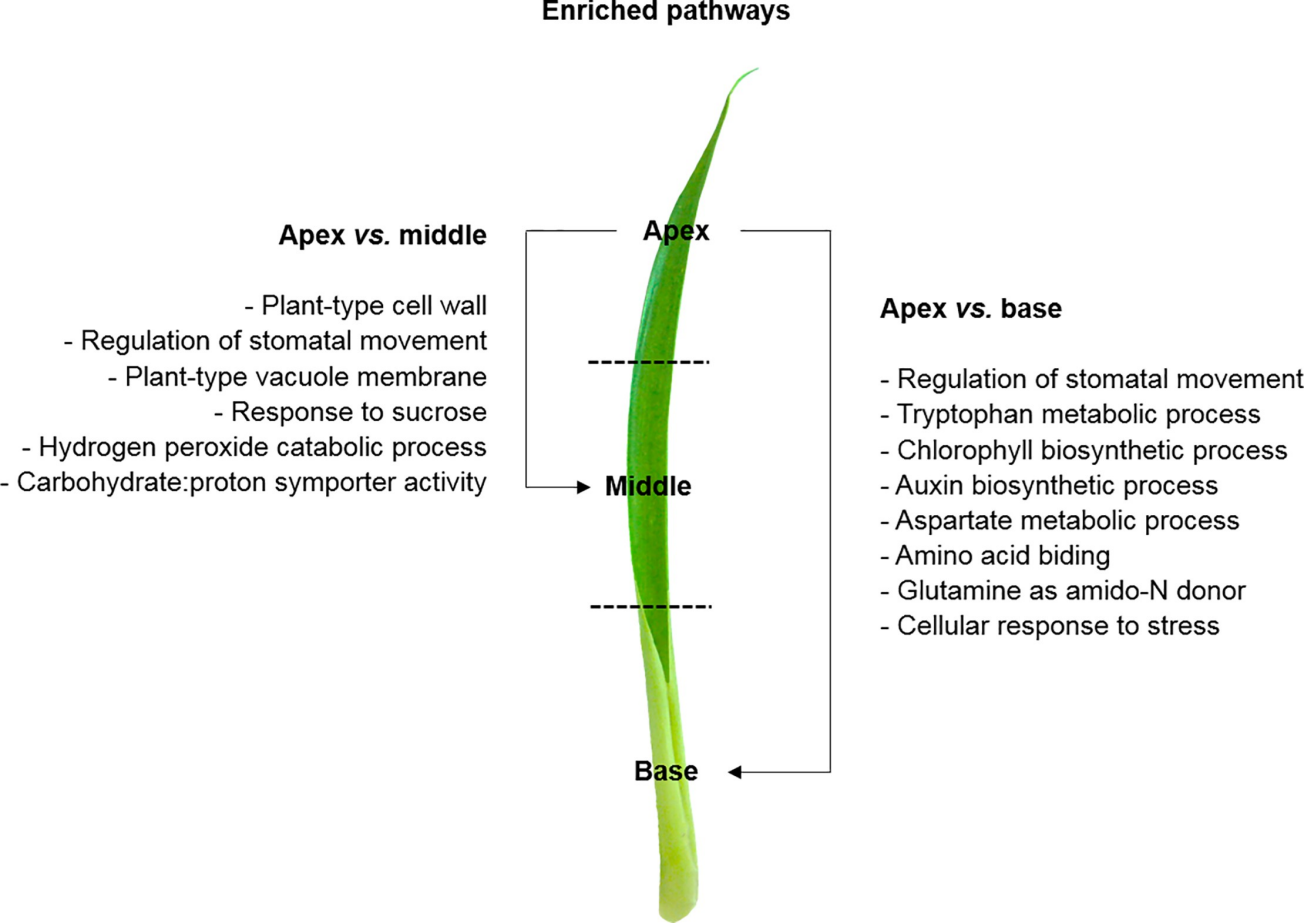
GO and DEGs of the apex leaf portion. Gene Ontology (GO) functional enriched pathways within differentially expressed genes (DEGs) between the apex and middle, and apex and base leaf portions of *Guzmania monostachia*. This figure was constructed based on the enriched pathways shown in S4 Fig (Supporting information), and according to the GO terms for biological process, cellular component, or molecular function categories, which showed differential abundance by Fisher’s exact test (FDR < 0.05).

**Fig 5:**
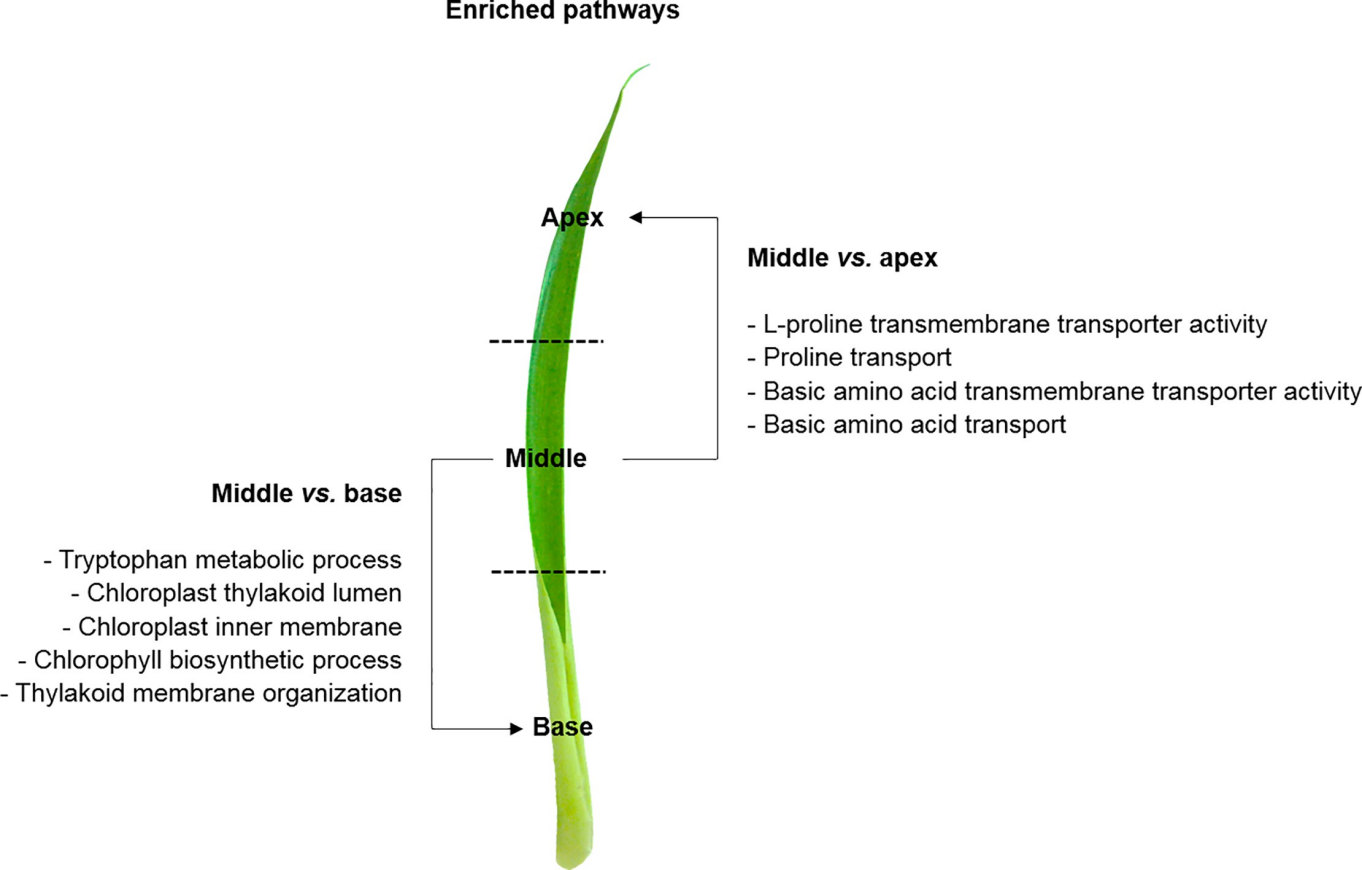
GO and DEGs of the middle leaf portion. Gene Ontology (GO) functional enriched pathways within differentially expressed genes (DEGs) between the middle and apex, and middle and base leaf portions of *Guzmania monostachia*. This figure was constructed based on the enriched pathways shown in S5 Fig (Supporting information), and according to the GO terms for biological process, cellular component, or molecular function categories, which showed differential abundance by Fisher’s exact test (FDR < 0.05).

**Fig 6:**
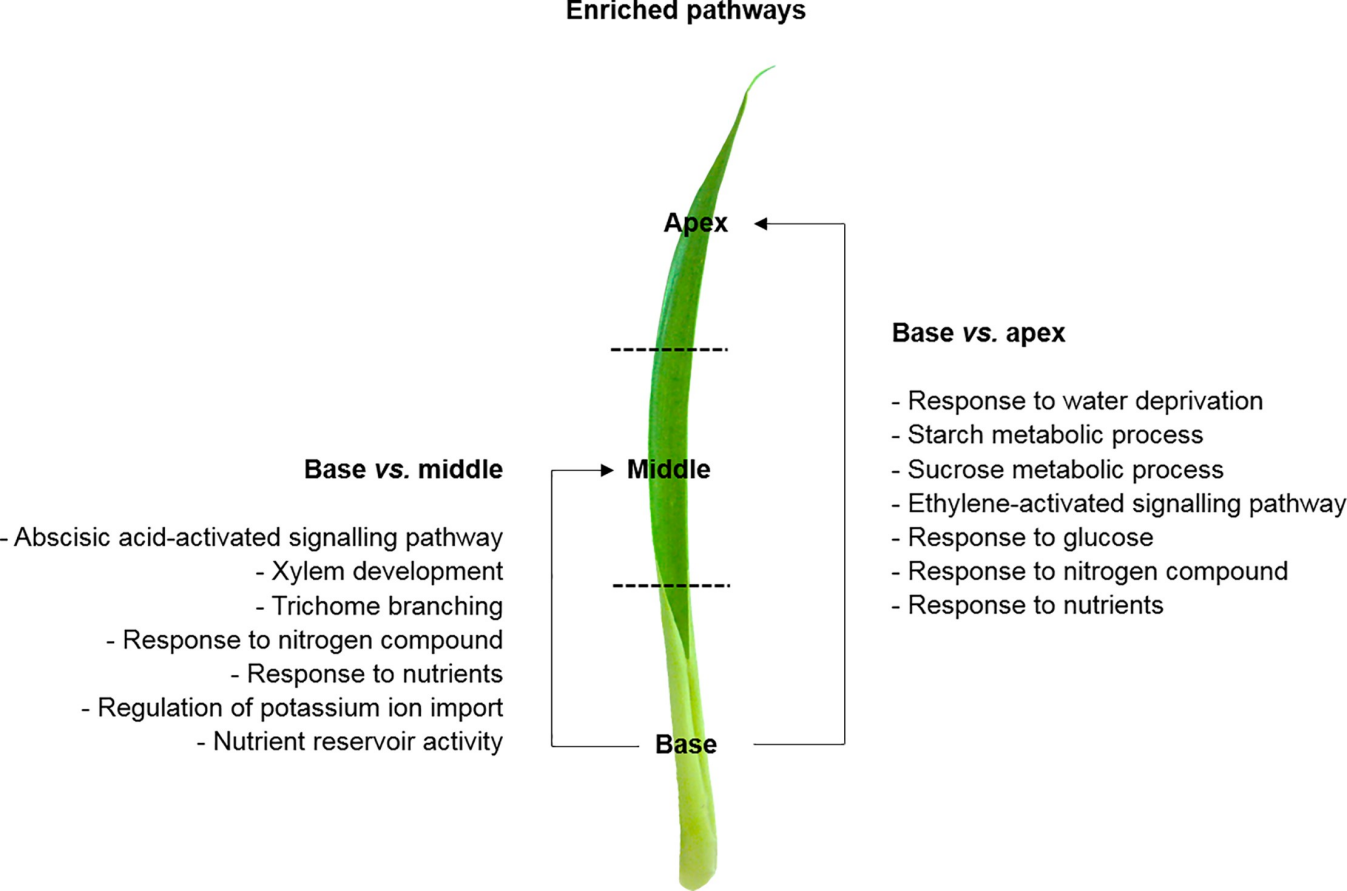
GO and DEGs of the base leaf portion. Gene Ontology (GO) functional enriched pathways within differentially expressed genes (DEGs) between the base and apex, and base and middle leaf portions of *Guzmania monostachia*. This figure was constructed based on the enriched pathways shown in S6 Fig (Supporting information), and according to the GO terms for biological process, cellular component, or molecular function categories, which showed differential abundance by Fisher’s exact test (FDR < 0.05).

Using the GO annotation, we also explored the most enriched pathways of each leaf portion through pair-wise comparisons. The most enriched pathways in the apex compared to the middle was associated with plant-type cell wall, regulation of stomatal movement, plant-type vacuole membrane, response to sucrose, carbohydrate:proton symporter activity, and hydrogen peroxide catabolic process (Fig 4, S4A Fig). Additionally, the most enriched pathways in the apex compared to the base was the regulation of stomatal movement, tryptophan metabolic process, chlorophyll biosynthetic process, auxin biosynthetic process, aspartate metabolic process, amino acid biding, glutamine as amido-N donor, and cellular response to stress (S2 and S3 Tables; Fig 4, S4B Fig). Furthermore, the most enriched pathways in the middle against the apex were L-proline transmembrane transporter activity, proline transport, basic amino acid transmembrane transporter activity (amino acid transporter, *AAP4*), and basic amino acid transport (S2 and S3 Tables; Fig 5, S5A Fig). On the other hand, the most enriched pathways in the middle against the base were tryptophan metabolic process, chloroplast thylakoid lumen, chloroplast inner membrane, chlorophyll biosynthesis process, and thylakoid membrane organization (S3 Table; Fig 5, S5B Fig). Finally, the most enriched pathways in the base compared to the apex was associated with response to water deprivation, starch and sucrose metabolic processes, ethylene-activated signaling pathway (ethylene biosynthesis, *ACCO1*), and response to glucose, nitrogen compound (nitrate transporter, *NRT1*.*1*) and nutrients (S2 and S4 Tables; Fig 6, S6A Fig). Compared to the middle portion, the most enriched pathways in the base were the abscisic acid-activated signaling pathway, xylem development, trichome branching, response to nitrogen compound and nutrients, regulation of potassium ion import (potassium transporter, *HAK26*; CBL interacting protein kinase, *CIPK23*), and nutrient reservoir activity (S2 and S4 Tables; Fig 6, S6B Fig).

## Discussion

The sequencing data of *Guzmania monostachia* represents the first transcriptome of a Tillandsioideae bromeliad whose subfamily traits are described as the most derived within the family. We highlight, for the first time, the differential gene expression among distinct bromeliad leaf portions, considering that the apex and the middle portions were very similar in their DEGs, and that both differed substantially from the base. Although the apex showed unigenes related especially to photosynthesis, oxidoreduction reactions, and cellular response to stress, unigenes of the leaf base showed an interesting association with the response to nutrients, trichome branching and response to water deprivation. Furthermore, unigenes of the middle portion was associated mainly with amino acid transport and amino acid transmembrane transporter functions.

Bromeliad leaves have been described as presenting a morpho-physiological gradient from the apex to the base [3–7]. Here, we demonstrate the genetic background behind this, since each leaf portion showed a set of unigenes associated with specific functions, despite the high similarity between the apex and middle portions. The apex of *G*. *monostachia* was functionally enriched concerning unigenes related to the photosynthesis, production of chlorophyll, regulation of stomatal movement and cellular response to stress (S2 and S3 Tables), which were coherent with the highest light intensity received by this portion and its higher density of stomata [4, 6]. In addition, it is in line with the higher expression of CAM compared to the leaf base showed by several papers [3, 5–7, 13–15], through the higher activity of the enzyme phosphoenolpyruvate carboxylase (PEPC) and higher nocturnal acidity accumulation. The apex of *G*. *monostachia* was also enriched regarding the processes of oxidoreduction reactions, and hydrogen peroxide catabolism especially with unigenes related to the expression of antioxidant enzymes, such as L-ascorbate peroxidase (S2 and S3 Tables). Accordingly, Abreu et al. [6] discussed that the highest CAM expression and light incidence in the leaf apex of *G*. *monostachia* have a direct influence on the hydrogen peroxide production. As a response to the ROS production, the apex has shown an enhanced activity of antioxidant enzymes like ascorbate peroxidase, possibly for photoprotection since the apex is more susceptible to photo-oxidative stress [6].

Despite the functions clearly performed by the apex and base portions described in previous studies, the roles of the middle leaf portion are still poorly studied. As a matter of fact, Takahashi and Mercier [4] showed in other epiphytic tank bromeliad that the apex and base have different enzymes related to nitrogen metabolism, such as the nitrate reductase that reduces nitrate to nitrite, which is converted to ammonium at the base. They hypothesized the ammonium translocation through the middle portion until the apex, where glutamine synthetase converts glutamate to glutamine using this translocated ammonium [4]. Here, we observed the enriched pathways and identified unigenes in the apex related to the carbon-N ligase activity with glutamine as amido-N donor, while the middle portion was enriched for basic amino acid transport, proline transport, amino acid transmembrane transporters, and proline transmembrane transporter (S2 and S3 Tables).

The leaf base of epiphytic tank bromeliads is exposed to the lowest luminosity due to its closely imbricated leaves in a rosette-forming tank, which are in contact with water and nutrients [27, 28]. Considering that the leaf base has a high density of trichomes [5, 15], this portion has been associated physiologically with the absorption of water and nutrients. In fact, we showed in the present study that some of the most enriched pathways performed by the leaf base were the response to nutrients, response to nitrogen compound, regulation of potassium ion import, and trichome branching (S2 and S3 Tables). Additionally, the base portion showed enriched pathways concerning the response to water deprivation, and signalling of hormones, such as ethylene and abscisic acid (ABA) (S2 and S3 Tables). An essential response to ethylene is the promotion of aerenchyma in O_2_ deprived tissues [29] and, interestingly, the basal leaf portion of *G*. *monostachia* which is in contact with the tank solution has the highest degree of specialization in its trichome structure [30] and large air channels arranged longitudinally and parallel to vascular bundles at the leaf base [15]. Rodrigues et al [15] also quantified the highest endogenous ABA level in the basal foliar portion in response to water shortage, which may explain why this leaf portion was enriched concerning the ABA signalling pathway.

In conclusion, our transcriptome results showed differentially expressed genes along the leaf blade of *G*. *monostachia*, which possibly occurs in many other bromeliad species. As a Tillandsioideae species whose subfamily is described to have the most derived traits within the family, DEGs from the apical leaf portion were mainly related to the regulation of stomatal movement, production of chlorophyll, cellular response to stress, and H_2_O_2_ catabolic process. In contrast, the middle portion showed DEGs associated with the transport of amino acids, while the DEGs from the leaf base were mainly linked with responses to nutrients, response to nitrogen compounds, regulation of potassium ion import, response to water deprivation and trichome branching suggesting that, at least in part, this leaf portion can replace some of the root function of terrestrial plants. We highlight that our transcriptome results can still be explored in future studies comparing DEGs in regards to different leaf portions, since we make available all reads of each leaf portion. Therefore, possibly candidate unigenes and numerous enriched pathways could be prospected in future experimental work, opening new possibilities to bioengineer non-inducible CAM plants and/or improve the fertilization use efficiency by increasing leaf nutrient acquisition of crop plants.

## Supporting information

S1 Table Reads of the apex, middle, and base leaf portions of *Guzmania monostachia*. A total of 54 samples were analysed (18 samples per leaf portion) and the reads mapped were used for differently expressed genes (DEGs) analyses. Reads are available at the Bioproject ID PRJNA532595.
(DOC)

S2 Table DEGs of each leaf portion of *Guzmania monostachia*. List of differently expressed genes (DEGs) related to the biological functions performed by each leaf portion [apex (A), middle (M), base (B)] of *Guzmania monostachia*. Genes of interest are compared according to their upregulation among each leaf portion; upregulation are expressed in log fold change (Log FC) and Fisher’s exact test (FDR < 0.05; in bold).
(DOC)

S3 Table GO and DEGs of the chlorophyll *vs*. less chlorophyll leaf portions. Gene Ontology (GO) functional enrichment within differentially expressed genes (DEGs) between the chlorophyll leaf portion (apex and middle) compared to the less chlorophyll portion (base) of *Guzmania monostachia* (apex *vs*. base, and middle *vs*. base). Each GO term presented the absolute number and percentage (in parenthesis) of the enriched unigenes in each leaf portion, as well as the number of the unigenes present in the functional annotation of the samplings used as references. The GO terms correspond to the biological process, cellular component, or molecular function categories, which showed differential abundance according to Fisher’s exact test (cut-off FDR < 0.001).
(DOC)

S4 Table GO and DEGs of the less chlorophyll *vs*. chlorophyll leaf portions. Gene Ontology (GO) functional enrichment within differentially expressed genes (DEGs) between the less chlorophyll portion (base) compared to the chlorophyll leaf portion (apex and middle) of *Guzmania monostachia* (base *vs*. apex, and base *vs*. middle). Each GO term presented the absolute number and percentage (in parenthesis) of the enriched unigenes in each leaf portion, as well as the number of the unigenes present in the functional annotation of the samplings used as references. The GO terms correspond to the biological process, cellular component, or molecular function categories, which showed differential abundance. according to Fisher’s exact test (cut-off FDR < 0.001).
(DOC)

S1 Fig PC analysis of apex *vs*. base. Principal component (PC) analysis of samples of *Guzmania monostachia* leaf portions (apex and base) submitted to different treatments (drought with nutrients, drought without nutrients, and received water but not nutrients). The PC analysis indicates the profiles of the samples: similar samples form clusters, while dissimilar samples are found at greater distances. The PC1 (85% of sample variation) is dividing the apex from the base.
(TIF)

S2 Fig PC analysis of apex *vs*. middle. Principal component (PC) analysis of samples of *Guzmania monostachia* leaf portions (apex and middle) submitted to different treatments (drought with nutrients, drought without nutrients, and received water but not nutrients). The PC analysis indicates the profiles of the samples: similar samples form clusters, while dissimilar samples are found at greater distances. The PC1 (86% of sample variation) is dividing samples, in which apex and middle were more similar than discrepant.
(TIF)

S3 Fig PC analysis of base *vs*. middle. Principal component (PC) analysis of samples of *Guzmania monostachia* leaf portions (base and middle) submitted to different treatments (drought with nutrients, drought without nutrients, and received water but not nutrients). The PC analysis indicates the profiles of the samples: similar samples form clusters, while dissimilar samples are found at greater distances. The PC1 (80% of sample variation) is dividing the base from the middle.
(TIF)

S4 Fig GO and DEGs of the apex leaf portion. Gene Ontology (GO) functional enrichment within differentially expressed genes (DEGs). The histograms represent a multilevel chart of the most specific GO terms for the biological process, cellular component, or molecular function categories, which showed differential abundance according to Fisher’s exact test (FDR < 0.05). Values are expressed as percentage of the total upregulated genes in the sample (test set; blue) compared to the total upregulated genes in all samples (reference set; red). Comparisons between **(A)** apex and middle, and **(B)** apex and base leaf portions of *Guzmania monostachia* are shown.
(TIF)

S5 Fig GO and DEGs of the middle leaf portion. Gene Ontology (GO) functional enrichment within differentially expressed genes (DEGs). The histograms represent a multilevel chart of the most specific GO terms for the biological process, cellular component, or molecular function categories, which showed differential abundance according to Fisher’s exact test (FDR < 0.05). Values are expressed as percentage of the total upregulated genes in the sample (test set; blue) compared to the total upregulated genes in all samples (reference set; red). Comparisons between **(A)** middle and apex, and **(B)** middle and base leaf portions of *Guzmania monostachia* are shown.
(TIF)

S6 Fig GO and DEGs of the base leaf portion. Gene Ontology (GO) functional enrichment within differentially expressed genes (DEGs). The histograms represent a multilevel chart of the most specific GO terms for the biological process, cellular component, or molecular function categories, which showed differential abundance according to Fisher’s exact test (FDR < 0.05). Values are expressed as percentage of the total upregulated genes in the sample (test set; blue) compared to the total upregulated genes in all samples (reference set; red). Comparisons between **(A)** base and apex, and **(B)** base and middle leaf portions of *Guzmania monostachia* are shown.
(TIF)
