# Supplementary material for: Transcriptional foliar profile of the C3-CAM bromeliad Guzmania monostachia
Source: PLoS One. 2019 Oct 29;14(10):e0224429. doi: 10.1371/journal.pone.0224429 (PMC6818958; doi:10.1371/journal.pone.0224429)
Supplement: S1 Table — A total of 54 samples were analysed (18 samples per leaf portion) and the reads mapped were used for differently expressed genes (DEGs) analyses. Reads are available at the Bioproject ID PRJNA532595. (DOC) [file pone.0224429.s001.doc]

**S1 Table. Reads of the apex, middle, and base leaf portions of *Guzmania monostachia*. A total of 54 samples were analysed (18 samples per leaf portion) and the reads mapped were used for differently expressed genes (DEGs) analyses. Reads are available at the Bioproject ID PRJNA532595**

| **Sample** | **Total number of reads** | **Number of reads after filtering** | **Number of reads uniquely mapped** |
| --- | --- | --- | --- |
| **Apex** |  |  |  |
| G1A | 14892990 | 12978640 | 27871630 |
| G1B | 14465006 | 12596675 | 27061681 |
| G1C | 13430492 | 11683788 | 25114280 |
| G4B | 13220155 | 11497492 | 24717647 |
| G4C | 12829931 | 11171148 | 24001079 |
| G4D | 13347217 | 11628857 | 24976074 |
| G7A | 13588600 | 11760785 | 25349385 |
| G7B | 12811939 | 11084306 | 23896245 |
| G7D | 13831931 | 11991860 | 25823791 |
| G10A | 16219087 | 14076278 | 30295365 |
| G10B | 14955069 | 12969006 | 27924075 |
| G10C | 14207310 | 12316173 | 26523483 |
| G13A | 14305051 | 12366660 | 26671711 |
| G13C | 13602602 | 11801748 | 25404350 |
| G13D | 13338144 | 11593354 | 24931498 |
| G16A | 13665287 | 11934441 | 25599728 |
| G16B | 14844840 | 12939882 | 27784722 |
| G16D | 11787175 | 10271763 | 22058938 |
| **Middle** |  |  |  |
| G2A | 14698274 | 12822051 | 27520325 |
| G2C | 13153688 | 11451395 | 24605083 |
| G2D | 13372755 | 11641508 | 25014263 |
| G5A | 14649463 | 12748795 | 27398258 |
| G5B | 12772553 | 11106845 | 23879398 |
| G5D | 13658844 | 11889720 | 25548564 |
| G8A | 13150063 | 11449233 | 24599296 |
| G8B | 13091273 | 11395404 | 24486677 |
| G8D | 13192577 | 11465666 | 24658243 |
| G11A | 14978489 | 13105223 | 28083712 |
| G11C | 16008434 | 13953510 | 29961944 |
| G11D | 14015261 | 12229415 | 26244676 |
| G14A | 15028450 | 13053373 | 28081823 |
| G14C | 14302256 | 12446978 | 26749234 |
| G14D | 12954848 | 11262264 | 24217112 |
| G17A | 14531370 | 12668629 | 27199999 |
| G17B | 13356485 | 11607753 | 24964238 |
| G17C | 13981713 | 12133709 | 26115422 |
| **Base** |  |  |  |
| G3A | 12554129 | 10867883 | 23422012 |
| G3C | 13460661 | 11655707 | 25116368 |
| G3D | 13156403 | 11437390 | 24593793 |
| G6A | 14201892 | 12279171 | 26481063 |
| G6B | 12866261 | 11096869 | 23963130 |
| G6C | 16634345 | 14306171 | 30940516 |
| G9A | 14543820 | 12560900 | 27104720 |
| G9C | 13462221 | 11576796 | 25039017 |
| G9D | 13415526 | 11575658 | 24991184 |
| G12A | 15003552 | 13115048 | 28118600 |
| G12B | 13684991 | 11936806 | 25621797 |
| G12C | 13539266 | 11787231 | 25326497 |
| G15B | 13863399 | 11971231 | 25834630 |
| G15C | 15498711 | 13380907 | 28879618 |
| G15D | 13672399 | 11995240 | 25667639 |
| G18A | 13287919 | 11435487 | 24723406 |
| G18B | 15733564 | 13557483 | 29291047 |
| G18D | 14163145 | 12229123 | 26392268 |
