# Supplementary material for: Transcriptional foliar profile of the C3-CAM bromeliad Guzmania monostachia
Source: PLoS One. 2019 Oct 29;14(10):e0224429. doi: 10.1371/journal.pone.0224429 (PMC6818958; doi:10.1371/journal.pone.0224429)
Supplement: S2 Table — List of differently expressed genes (DEGs) related to the biological functions performed by each leaf portion [apex (A), middle (M), base (B)] of Guzmania monostachia. Genes of interest are compared according to their upregulation among each leaf portion; upregulation are expressed in log fold change (Log FC) and Fisher’s exact test (FDR < 0.05; in bold). (DOC) [file pone.0224429.s002.doc]

**S2 Table. DEGs of each leaf portion of *Guzmania monostachia*. List of differently expressed genes (DEGs) related to the biological functions performed by each leaf portion [apex (A), middle (M), base (B)] of *Guzmania monostachia*. Genes of interest are compared according to their upregulation among each leaf portion; upregulation are expressed in log fold change (Log FC) and Fisher’s exact test (FDR < 0.05; in bold)**

| **(Unigenes) Homolog genes** | **Comparisons among leaf portions** | **Log FC** | ***P*** |
| --- | --- | --- | --- |
| (c20928_g1_i8) Chlorophyll synthase (*CHLG*) [*Arabidopsis thaliana*] | Upregulated in Apex (A *vs.* B)  Upregulated in Middle (M *vs.* B) | 0.802  -0.872 | **< 0.001**  **< 0.001** |
| (c12580_g1_i1) Soluble starch synthase (*SSY1*) [*Oryza sativa*] | Upregulated in Apex (A *vs.* B)  Upregulated in Middle (M *vs.* B) | 2.483  -2.352 | **< 0.001**  **0.001** |
| (c22549_g1_i2) Phosphoenolpyruvate carboxylase (*PEPC1*) [*Sorghum bicolor*] | Upregulated in Apex (A *vs.* B) | 0.620 | **<0.001** |
| (c8806_g1_i2) Ascorbate peroxidase (*APX4*) [*Oryza sativa*] | Upregulated in Apex (A *vs.* B) | 1.335 | **<0.001** |
| (c13260_g1_i1) Amino acid transporter (*AAP4*) [*Arabidopsis thaliana*] | Upregulated in Middle (M *vs.* A) | -1.101 | **<0.001** |
| (c19679_g1_i6) Nitrate transporter (*NRT1.1*) [*Arabidopsis thaliana*] | Upregulated in Base (B *vs.* M) | -0.647 | **<0.001** |
| (c20907_g1_i5) Potassium transporter (*HAK26*) [*Oryza sativa*] | Upregulated in Base (B *vs.* M) | 2.714 | **<0.001** |
| (c22128_g1_i7) CBL interacting protein kinase (*CIPK23*) [*Oryza sativa*] | Upregulated in Base (B *vs.* M) | 1.070 | **0.006** |
| (c18037_g2_i1) Cellulose synthase (*CESA9*) [*Oryza sativa*] | Upregulated in Base (B *vs.* A)  Upregulated in Base (B *vs.* M) | -5.752  5.638 | **<0.001**  **<0.001** |
| (c738_g1_i2) Jasmonic acid biosynthesis (*LOX4*) [*Oryza sativa*] | Upregulated in Base (B *vs.* A)  Upregulated in Base (B *vs.* M) | -3.074  2.005 | **<0.001**  **<0.001** |
| (c32058_g1_i1) Ethylene biosynthesis (*ACCO1*) [*Solanum lycopersicum*] | Upregulated in Base (B *vs.* M) | 3.325 | **<0.001** |
