# Supplementary material for: Transcriptional foliar profile of the C3-CAM bromeliad Guzmania monostachia
Source: PLoS One. 2019 Oct 29;14(10):e0224429. doi: 10.1371/journal.pone.0224429 (PMC6818958; doi:10.1371/journal.pone.0224429)
Supplement: S3 Table — Gene Ontology (GO) functional enrichment within differentially expressed genes (DEGs) between the chlorophyll leaf portion (apex and middle) compared to the less chlorophyll portion (base) of Guzmania monostachia (apex vs. base, and middle vs. base). Each GO term presented the absolute number and percentage (in parenthesis) of the enriched unigenes in each leaf portion, as well as the number of the unigenes present in the functional annotation of the samplings used as references. The GO terms correspond to the biological process, cellular component, or molecular function categories, which showed differential abundance according to Fisher’s exact test (cut-off FDR < 0.001). (DOC) [file pone.0224429.s003.doc]

**S3 Table. GO and DEGs of the chlorophyll *vs.* less chlorophyll leaf portions. Gene Ontology (GO) functional enrichment within differentially expressed genes (DEGs) between the chlorophyll leaf portion (apex and middle) compared to the less chlorophyll portion (base) of *Guzmania monostachia* (apex *vs.* base, and middle *vs.* base). Each GO term presented the absolute number and percentage (in parenthesis) of the enriched unigenes in each leaf portion, as well as the number of the unigenes present in the functional annotation of the samplings used as references. The GO terms correspond to the biological process, cellular component, or molecular function categories, which showed differential abundance according to Fisher’s exact test (cut-off FDR < 0.001)**

| **GO ID** | **GO description** | **Unigenes assigned** | | | |
| --- | --- | --- | --- | --- | --- |
|  |  | **Apex *vs.* base** | **Reference** | **Middle *vs.* base** | **Reference** |
| **GO:0015979** | Photosynthesis | 179/6952 | 195/37510 | 172/6681 | 202/37781 |
|  |  | (2.57) | (0.52) | (2.13) | (0.53) |
| **GO:0019684** | Photosynthesis, light reaction | 204/7837 | 322/27245 | 221/7942 | 305/27140 |
|  |  | (2.60) | (1.18) | (2.78) | (1.12) |
| **GO:0015995** | Chlorophyll biosynthetic process | 99/7942 | 189/27378 | 103/8060 | 185/27260 |
|  |  | (1.24) | (0.69) | (1.27) | (0.67) |
| **GO:0055114** | Oxidation-reduction process | 790/7251 | 2061/25506 | 808/7355 | 2043/25402 |
|  |  | (10.89) | (8.08) | (10.98) | (8.04) |
| **GO:0042440** | Pigment metabolic process | 301/7740 | 485/27082 | 299/7864 | 487/26958 |
|  |  | (3.88) | (1.79) | (3.80) | (1.80) |
| **GO:0019252** | Starch biosynthetic process | 49/7992 | 69/27498 | 49/8114 | 69/27376 |
|  |  | (0.61) | (0.25) | (0.60) | (0.25) |
| **GO:0008299** | Isoprenoid biosynthetic process | 149/7892 | 321/27246 | 143/8020 | 327/27118 |
|  |  | (1.88) | (1.17) | (1.78) | (1.20) |
| **GO:0016688** | L-ascorbate peroxidase activity | 21/8020 | 17/27550 | 21/8142 | 17/27428 |
|  |  | (0.26) | (0.06) | (0.25) | (0.06) |
| **GO:0016884** | Carbon-N ligase activity, with | 21/8020 | 20/27547 | - | - |
|  | glutamine as amido-N donor | (0.26) | (0.07) |  |  |
| **GO:0033554** | Cellular response to stress | 774/7267 | 2278/25289 | - | - |
|  |  | (10.65) | (9.00) |  |  |
