# Supplementary material for: Transcriptional foliar profile of the C3-CAM bromeliad Guzmania monostachia
Source: PLoS One. 2019 Oct 29;14(10):e0224429. doi: 10.1371/journal.pone.0224429 (PMC6818958; doi:10.1371/journal.pone.0224429)
Supplement: S4 Table — Gene Ontology (GO) functional enrichment within differentially expressed genes (DEGs) between the less chlorophyll portion (base) compared to the chlorophyll leaf portion (apex and middle) of Guzmania monostachia (base vs. apex, and base vs. middle). Each GO term presented the absolute number and percentage (in parenthesis) of the enriched unigenes in each leaf portion, as well as the number of the unigenes present in the functional annotation of the samplings used as references. The GO terms correspond to the biological process, cellular component, or molecular function categories, which showed differential abundance. according to Fisher’s exact test (cut-off FDR < 0.001). (DOC) [file pone.0224429.s004.doc]

**S4 Table. GO and DEGs of the less chlorophyll *vs.* chlorophyll leaf portions. Gene Ontology (GO) functional enrichment within differentially expressed genes (DEGs) between the less chlorophyll portion (base) compared to the chlorophyll leaf portion (apex and middle) of *Guzmania monostachia* (base *vs.* apex, and base *vs.* middle). Each GO term presented the absolute number and percentage (in parenthesis) of the enriched unigenes in each leaf portion, as well as the number of the unigenes present in the functional annotation of the samplings used as references. The GO terms correspond to the biological process, cellular component, or molecular function categories, which showed differential abundance according to Fisher’s exact test (cut-off FDR < 0.001)**

| **GO ID** | **GO description** | **Unigenes assigned** | | | |
| --- | --- | --- | --- | --- | --- |
|  |  | **Base *vs.* apex** | **Reference** | **Base *vs.* middle** | **Reference** |
| **GO:1901698** | Response to nitrogen compound | 417/7888 | 948/26355 | 418/7665 | 947/26578 |
|  |  | (5.28) | (3.59) | (5.45) | (3.56) |
| **GO:0007584** | Response to nutrient | 71/8234 | 108/27195 | 81/8002 | 98/27427 |
|  |  | (0.86) | (0.39) | (1.01) | (0.35) |
| **GO:0009753** | Response to jasmonic acid | 369/7936 | 712/26591 | 346/7737 | 735/26790 |
|  |  | (4.64) | (2.67) | (4.47) | (2.74) |
| **GO:0009873** | Ethylene-activated signaling pathway | 114/8191 | 267/27036 | 117/7966 | 264/27261 |
|  |  | (1.39) | (0.98) | (1.46) | (0.96) |
| **GO:0006633** | Fatty acid biosynthetic process | 168/8137 | 330/26973 | 151/7932 | 347/27178 |
|  |  | (2.06) | (1.22) | (1.90) | (1.27) |
| **GO:0009664** | Plant-type cell wall organization | 384/7921 | 528/26775 | 207/7876 | 275/27250 |
|  |  | (4.84) | (1.97) | (2.62) | (1.00) |
| **GO:0030244** | Cellulose biosynthetic process | 101/8204 | 125/27178 | 104/7979 | 122/27403 |
|  |  | (1.23) | (0.45) | (1.30) | (0.44) |
| **GO:0010091** | Trichome branching | 74/8231 | 107/27196 | - | - |
|  |  | (0.89) | (0.39) |  |  |
| **GO:0043266** | Regulation of potassium ion import | - | - | 45/8038 | 76/27449 |
|  |  |  |  | (0.55) | (0.27) |
| **GO:0045735** | Nutrient reservoir activity | - | - | 20/8063 | 27/27498 |
|  |  |  |  | (0.24) | (0.09) |
