# Supplementary figures and images for: Transcriptional foliar profile of the C3-CAM bromeliad Guzmania monostachia
Source: PLoS One. 2019 Oct 29;14(10):e0224429. doi: 10.1371/journal.pone.0224429 (PMC6818958; doi:10.1371/journal.pone.0224429)

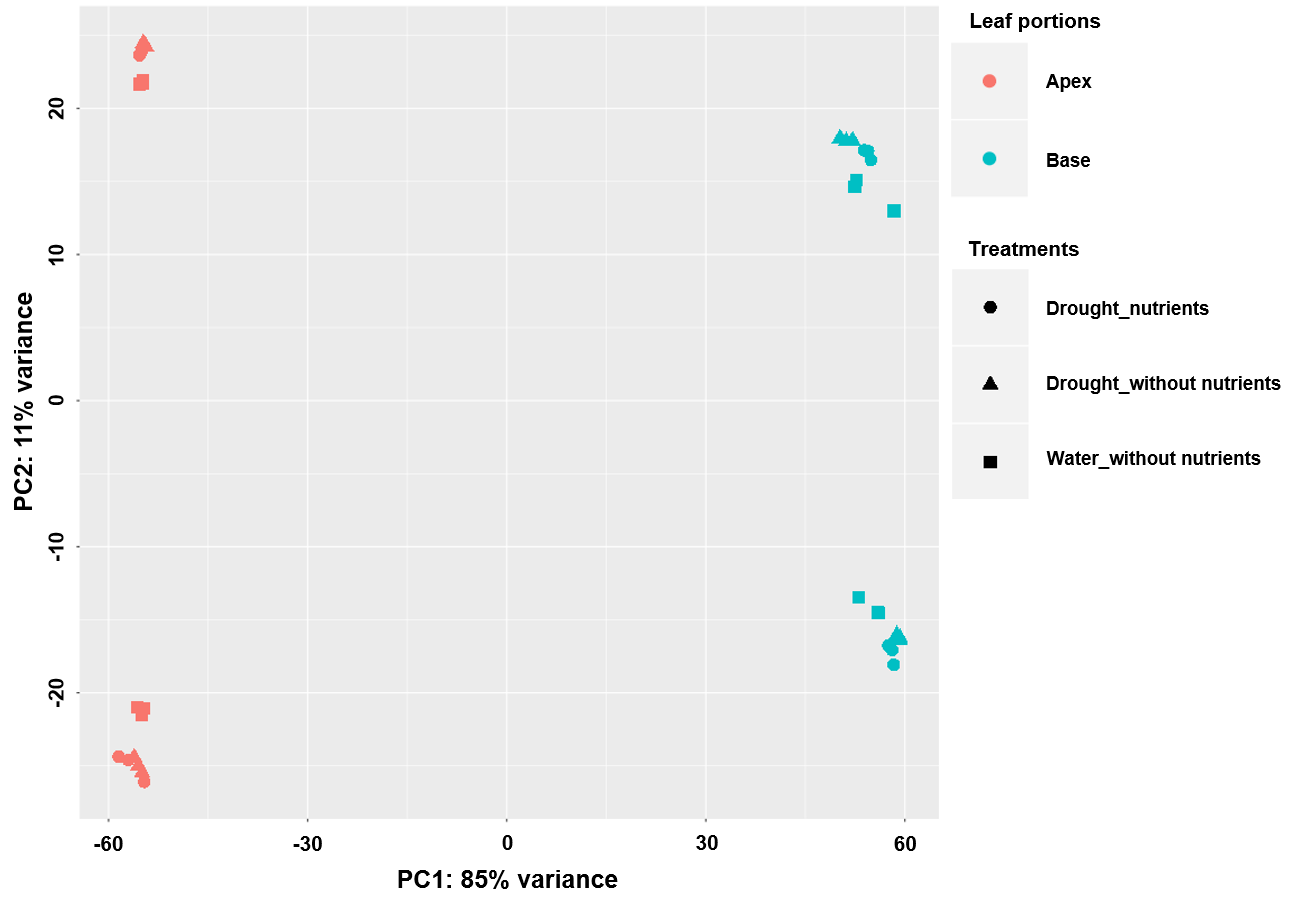

Supplement: S1 Fig — Principal component (PC) analysis of samples of Guzmania monostachia leaf portions (apex and base) submitted to different treatments (drought with nutrients, drought without nutrients, and received water but not nutrients). The PC analysis indicates the profiles of the samples: similar samples form clusters, while dissimilar samples are found at greater distances. The PC1 (85% of sample variation) is dividing the apex from the base. (TIF) [file pone.0224429.s005.tif]

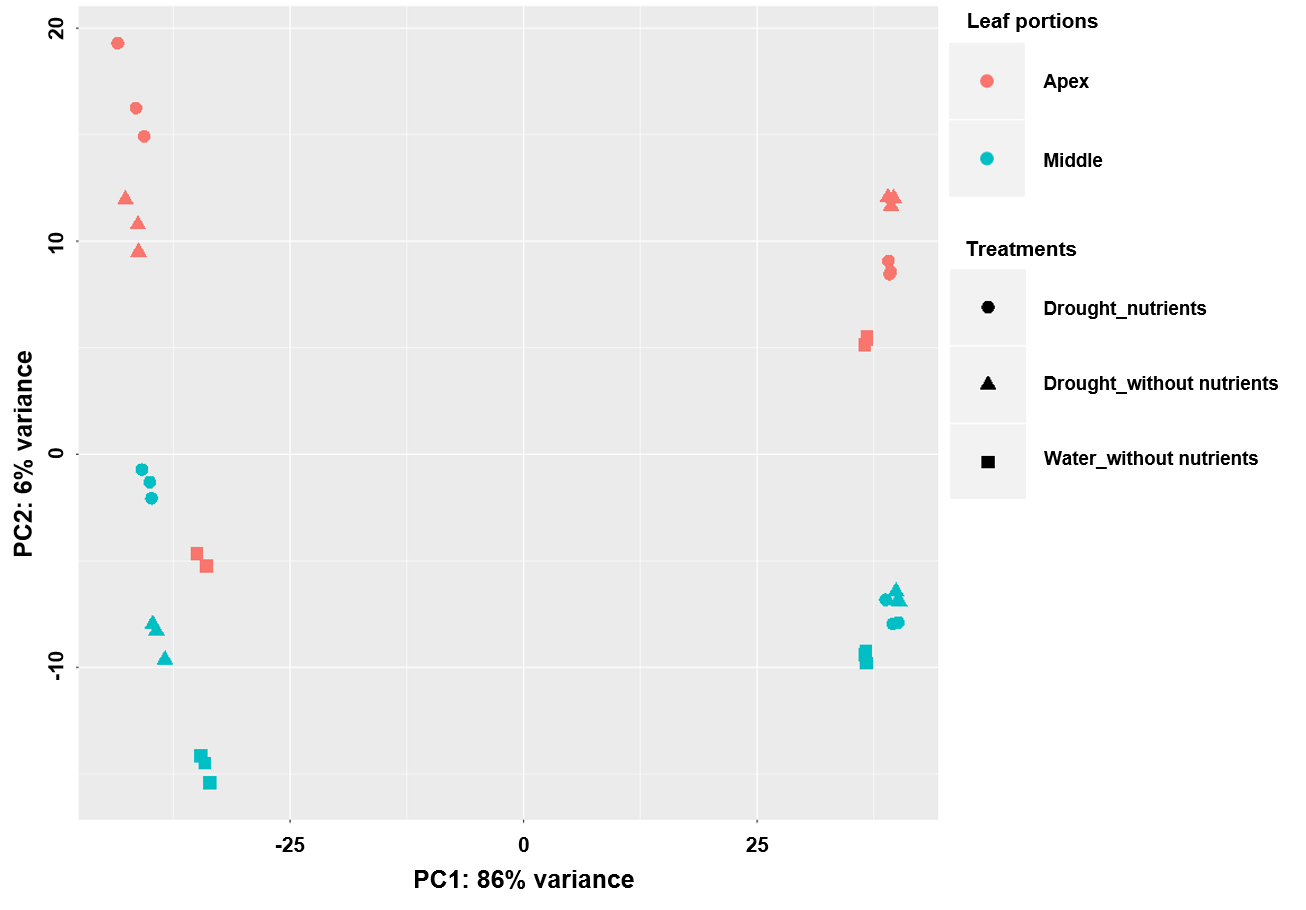

Supplement: S2 Fig — Principal component (PC) analysis of samples of Guzmania monostachia leaf portions (apex and middle) submitted to different treatments (drought with nutrients, drought without nutrients, and received water but not nutrients). The PC analysis indicates the profiles of the samples: similar samples form clusters, while dissimilar samples are found at greater distances. The PC1 (86% of sample variation) is dividing samples, in which apex and middle were more similar than discrepant. (TIF) [file pone.0224429.s006.tif]

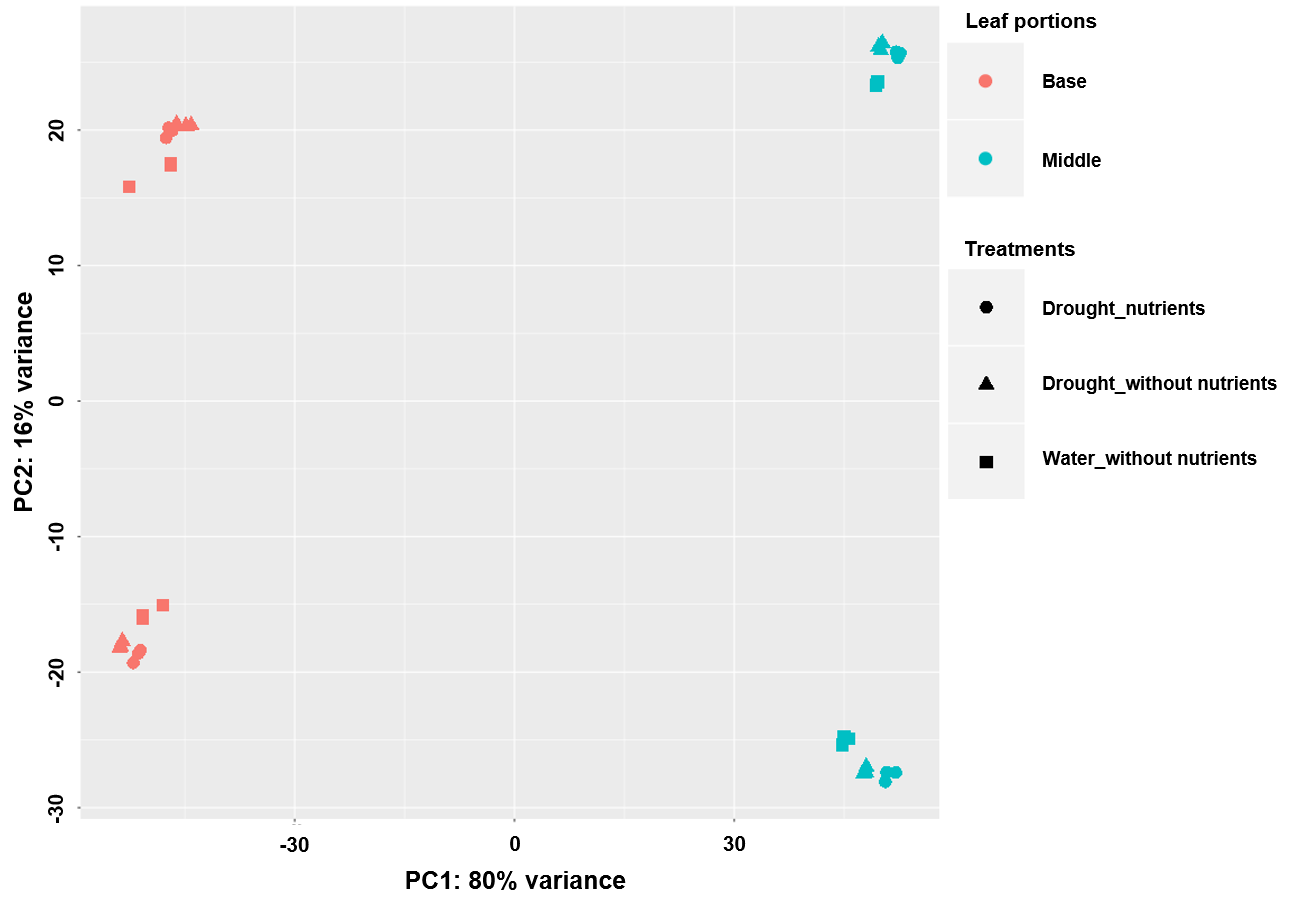

Supplement: S3 Fig — Principal component (PC) analysis of samples of Guzmania monostachia leaf portions (base and middle) submitted to different treatments (drought with nutrients, drought without nutrients, and received water but not nutrients). The PC analysis indicates the profiles of the samples: similar samples form clusters, while dissimilar samples are found at greater distances. The PC1 (80% of sample variation) is dividing the base from the middle. (TIF) [file pone.0224429.s007.tif]

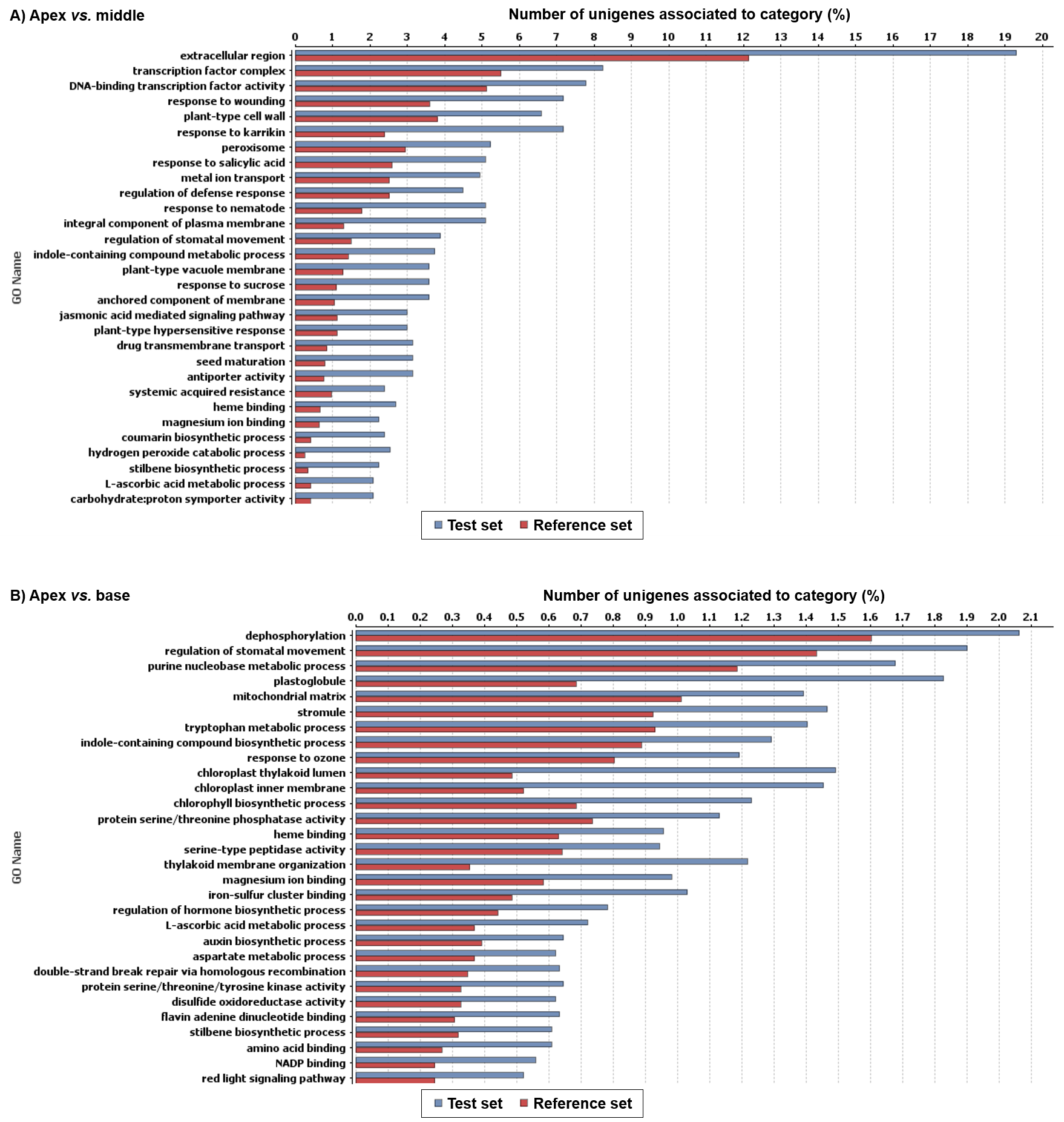

Supplement: S4 Fig — Gene Ontology (GO) functional enrichment within differentially expressed genes (DEGs). The histograms represent a multilevel chart of the most specific GO terms for the biological process, cellular component, or molecular function categories, which showed differential abundance according to Fisher’s exact test (FDR < 0.05). Values are expressed as percentage of the total upregulated genes in the sample (test set; blue) compared to the total upregulated genes in all samples (reference set; red). Comparisons between (A) apex and middle, and (B) apex and base leaf portions of Guzmania monostachia are shown. (TIF) [file pone.0224429.s008.tif]

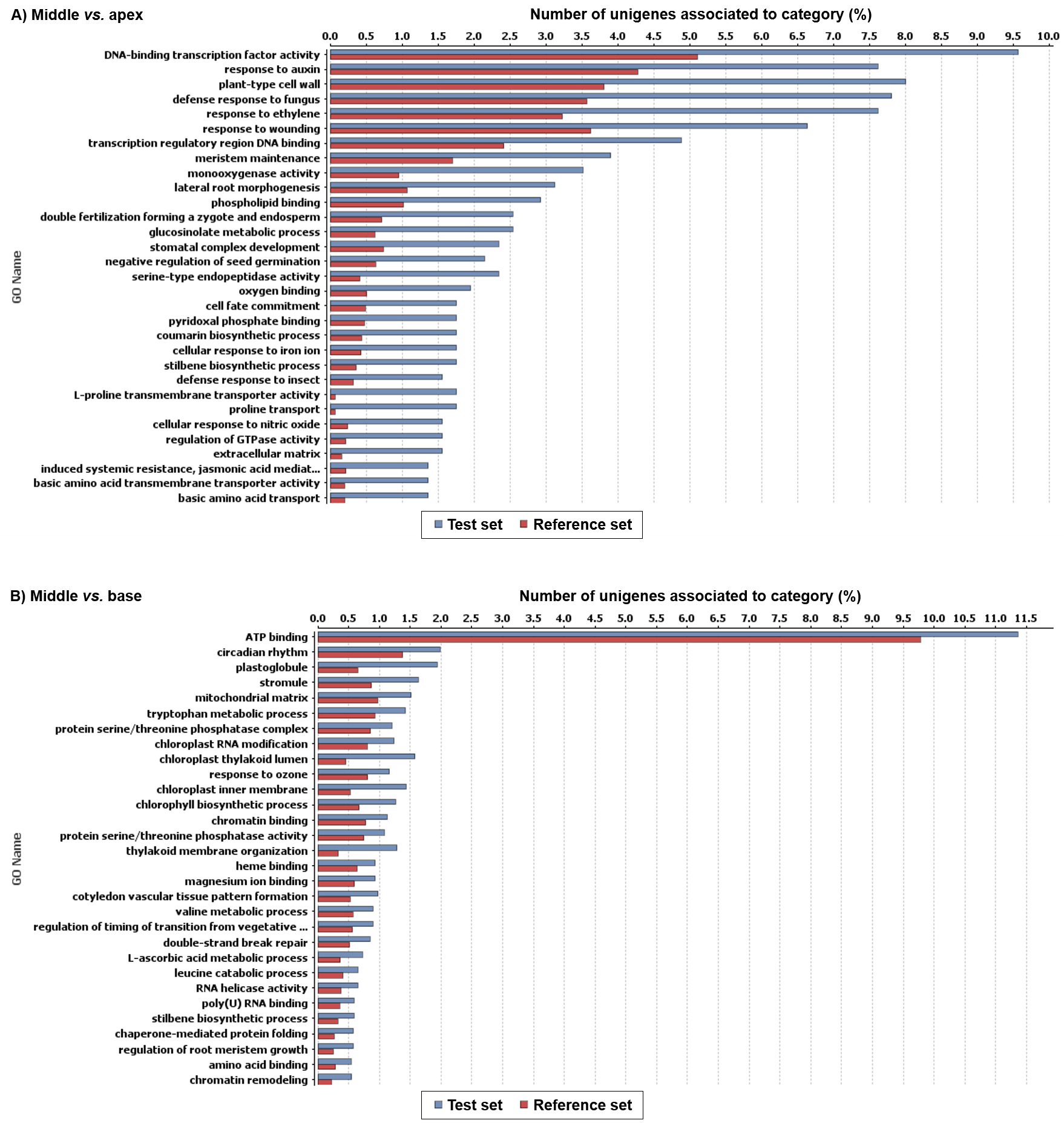

Supplement: S5 Fig — Gene Ontology (GO) functional enrichment within differentially expressed genes (DEGs). The histograms represent a multilevel chart of the most specific GO terms for the biological process, cellular component, or molecular function categories, which showed differential abundance according to Fisher’s exact test (FDR < 0.05). Values are expressed as percentage of the total upregulated genes in the sample (test set; blue) compared to the total upregulated genes in all samples (reference set; red). Comparisons between (A) middle and apex, and (B) middle and base leaf portions of Guzmania monostachia are shown. (TIF) [file pone.0224429.s009.tif]

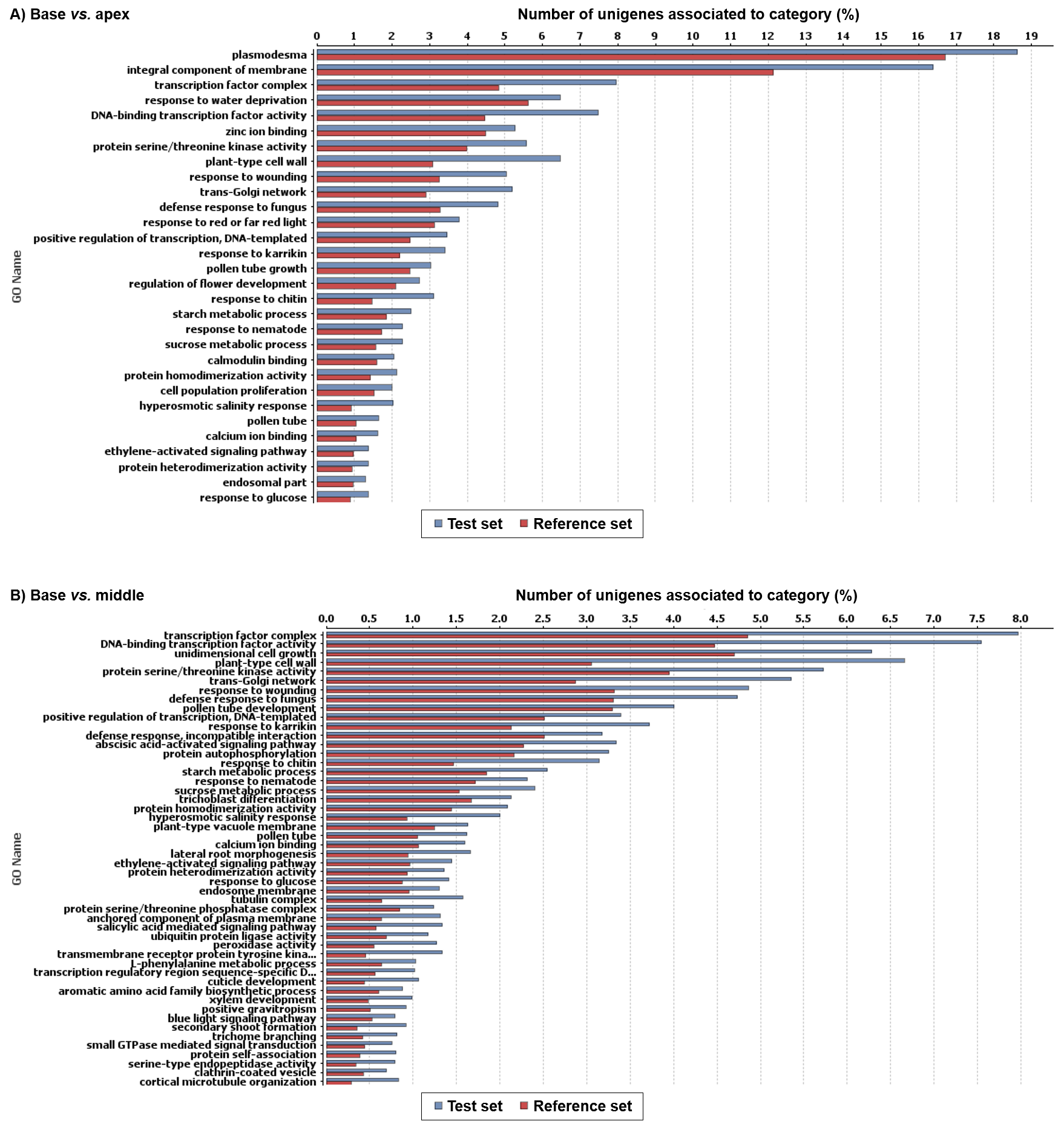

Supplement: S6 Fig — Gene Ontology (GO) functional enrichment within differentially expressed genes (DEGs). The histograms represent a multilevel chart of the most specific GO terms for the biological process, cellular component, or molecular function categories, which showed differential abundance according to Fisher’s exact test (FDR < 0.05). Values are expressed as percentage of the total upregulated genes in the sample (test set; blue) compared to the total upregulated genes in all samples (reference set; red). Comparisons between (A) base and apex, and (B) base and middle leaf portions of Guzmania monostachia are shown. (TIF) [file pone.0224429.s010.tif]
